# Role of interferon therapy in severe COVID-19: the COVIFERON randomized controlled trial

**DOI:** 10.1038/s41598-021-86859-y

**Published:** 2021-04-13

**Authors:** Ilad Alavi Darazam, Shervin Shokouhi, Mohamad Amin Pourhoseingholi, Seyed Sina Naghibi Irvani, Majid Mokhtari, Minoosh Shabani, Mahdi Amirdosara, Parham Torabinavid, Maryam Golmohammadi, SayedPayam Hashemi, Arsalan Azimi, Mohammad Hossein Jafarazadeh Maivan, Omidvar Rezaei, Alireza Zali, Mohammadreza Hajiesmaeili, Hadiseh Shabanpour Dehbsneh, Akram Hoseyni Kusha, Maryam Taleb Shoushtari, Negar Khalili, Azam Soleymaninia, Latif Gachkar, Ali Khoshkar

**Affiliations:** 1 Department of Infectious Diseases, Loghman Hakim Hospital, Shahid Beheshti University of Medical Sciences, Tehran, Iran; 2 Infectious Diseases and Tropical Medicine Research Center, Shahid Beheshti University of Medical Sciences, Tehran, Iran; 3 Basic and Molecular Epidemiology of Gastrointestinal Disorders Research Center, Research Institute for Gastroenterology and Liver Diseases, Shahid Beheshti University of Medical Sciences, Tehran, Iran; 4 Department of Pulmonary and Critical Care Medicine, Loghman Hakim Hospital, Shahid Beheshti University of Medical Sciences, Tehran, Iran; 5 Anesthesiology Research Center Loghman Hakim Hospital, Shahid Beheshti University of Medical Sciences, Tehran, Iran; 6 Student Research Committee, Hamedan University of Medical Sciences, Hamedan, Iran; 7 Shiraz University of Medical Sciences, Shiraz, Iran; 8 Clinical Research Development Unit, Bohlool Hospital, School of Medicine, Gonabad University of Medical Sciences, Gonabad, Iran; 9 Skull Base Research Center, Loghman Hakim Hospital, Shahid Beheshti University of Medical Sciences, Tehran, Iran; 10 Functional Neurosurgery Research Center, Shohada Tajrish Neurosurgical Center of Excellence, Shohada Tajrish Hospital, Shahid Beheshti University of Medical Sciences, Tehran, Iran; 11 Department of Surgery, Loghman Hakim Hospital, Shahid Beheshti University of Medical Sciences, Tehran, Iran; 12 SBMU Taskforce on The COVIFERON Study, Shahid Beheshti University of Medical Sciences, Tehran, Iran

**Keywords:** Infectious diseases, Respiratory tract diseases

## Abstract

Type 1 Interferons (IFNs) have been associated with positive effects on Coronaviruses. Previous studies point towards the superior potency of IFNβ compared to IFNα against viral infections. We conducted a three-armed, individually-randomized, open-label, controlled trial of IFNβ1a and IFNβ1b, comparing them against each other and a control group. Patients were randomly assigned in a 1:1:1 ratio to IFNβ1a (subcutaneous injections of 12,000 IU on days 1, 3, 6), IFNβ1b (subcutaneous injections of 8,000,000 IU on days 1, 3, 6), or the control group. All three arms orally received Lopinavir/Ritonavir (400 mg/100 mg twice a day for ten days) and a single dose of Hydroxychloroquine 400 mg on the first day. Our utilized primary outcome measure was Time To Clinical Improvement (TTCI) defined as the time from enrollment to discharge or a decline of two steps on the clinical seven-step ordinal scale, whichsoever came first. A total of 60 severely ill patients with positive RT-PCR and Chest CT scans underwent randomization (20 patients to each arm). In the Intention-To-Treat population, IFNβ1a was associated with a significant difference against the control group, in the TTCI; (HR; 2.36, 95% CI 1.10–5.17, P-value = 0.031) while the IFNβ1b indicated no significant difference compared with the control; HR; 1.42, (95% CI 0.63–3.16, P-value = 0.395). The median TTCI for both of the intervention groups was five days vs. seven days for the control group. The mortality was numerically lower in both of the intervention groups (20% in the IFNβ1a group and 30% in the IFNβ1b group vs. 45% in the control group). There were no significant differences between the three arms regarding the adverse events. In patients with laboratory-confirmed SARS-CoV-2 infection, as compared with the base therapeutic regiment, the benefit of a significant reduction in TTCI was observed in the IFNβ1a arm. This finding needs further confirmation in larger studies.

Trial Registration Number: ClinicalTrials.gov, NCT04343768. (Submitted: 08/04/2020; First Online: 13/04/2020) (Registration Number: NCT04343768).

## Introduction

In the last days of 2019, a group of mysterious pneumonia cases was communicated from Wuhan, China. One month later, the World Health Organization (WHO) unraveled the mystery a bit, and entitled the condition “Coronavirus Disease 2019” (COVID-19); furthermore, the International Committee on Taxonomy of Viruses (ICTV) designated the responsible virus as “Severe Acute Respiratory Syndrome Coronavirus 2” (SARS-CoV-2). Sweeping the world across, it was finally recognized as a “Public Health Emergency of International Concern” (PHEIC), and it did not take long before it was announced as a “Pandemic”^1,2^. Although symptoms of COVID-19 could vary from mild flu-like to respiratory compromise and multiorgan failure, most cases do not experience the severe form of the disease. However, because of the high level of contagiousness, COVID-19 has caused unparalleled global morbidity and mortality. Having spared only a few countries, SARS-CoV-2 has infected nearly 12 million people worldwide and has claimed over 500 thousand lives, as of July 7, 2020. These numbers are likely to only be underestimates^3–5^. Despite extensive global efforts, there are still no proven therapeutic options to combat this disease^6^.

Recently, United States Food and Drug Administration (FDA) issued an Emergency Use Authorization (EUA) for Remdesivir (GS-5734) based on a preliminary analysis of the topline data from a trial conducted by the National Institutes of Health (NIH). However, the existing data are far from conclusive as Yeming Wang and colleagues’ well-designed and meticulously conducted trial showed a numerically higher mortality rate in the Remdesivir arm compared to the placebo group, although the difference was not statistically significant. They also failed to show any significant benefit of the drug on Time To Clinical Improvement (TTCI) as their primary outcome measure, but these results should be interpreted with extreme caution as their study was underpowered^7,8^.

Hydroxychloroquine (HCQ) is another controversial therapeutic option that also has an EUA and has sparked a great deal of scientific and public debate. Despite showing early promise, more recent studies with better design have failed to show any consistent benefit from the drug^9–11^. Additionally a phase IIb Randomized Clinical Trial was terminated prematurely due to serious safety concerns of the higher dosages of HCQ^12^.

Bin Cao and colleagues revealed that Lopinavir/Ritonavir, another repurposed potential treatment option, albeit having an acceptable safety profile, lacked any significant efficacy in severe COVID-19 patients^13^.

Given the contradicting evidence on even the most promising pharmacologic treatments, more robust data are needed to uncover a much-needed effective therapy^6^.

SARS-CoV-2, a Betacoronavirus, shares most of its genes with the other two previously known deadly coronaviruses; the Middle East Respiratory Syndrome Coronavirus (MERS-CoV) and the Severe Acute Respiratory Syndrome Coronavirus (SARS-CoV)^2^. In addition, excessive, dysregulated and destructive inflammation is an essential common clinical characteristic of Middle East Respiratory Syndrome (MERS), Severe Acute Respiratory Syndrome (SARS) and COVID-19^14–16^. Consequently, it is suggested that, as in cases of SARS and MERS, regulators and modulators of the immune response, such as Interferons (IFNs), may perhaps alleviate pathogenesis of SARS-CoV-2^14,15,17–19^.

IFNs are natural antiviral and immune-modulating agents that initially react to viral infections and determine the ensuing course of the immune response to the infection. It has been shown that in the course of SARS and MERS, expression, and subsequently, the functions of Type I IFNs are markedly suppressed, and administration of exogenous Type I IFNs, is shown to reduce the severity of the symptoms of these diseases. Among all assessed Type I IFN products, various studies, including a systematic review, have indicated that IFN-β is far more potent than IFN-α as a coronavirus inhibitor. Furthermore, Interferon Beta-1b (IFNβ1b) and Interferon Beta-1a (IFNβ1a), were shown to have the most potent inhibitory effects on MERS-CoV and SARS-CoV^16,18–23^.

In this regard, we designed the current study to determine any possible effects and safety concerns of the two most promising exogenously administrable IFNs on the course and outcomes of patients hospitalized with severe COVID-19.

## Methods

### Trial design and oversight

We conducted the COVIFERON trial as an investigator-initiated, three-armed, parallel-group, individually-randomized, open-labeled, controlled trial for evaluation of the safety and efficacy of IFNβ1a and IFNβ1b versus an active control group in severe COVID-19 patients admitted to a major referral medical center in Tehran, Iran.

We randomly assigned eligible patients with confirmed SARS-Cov-2 infections to one of the three following therapeutic regimens: (1) IFNβ1a (Recigen) (Subcutaneous injections of 44 µg (12,000 IU) on days 1, 3, 6) + Hydroxychloroquine + Lopinavir/Ritonavir (Kaletra) [IFNβ1a group], (2) IFNβ1b (Ziferon) (Subcutaneous injections of 0.25 mg (8,000,000 IU) on days 1, 3, 6) + Hydroxychloroquine + Lopinavir/Ritonavir (Kaletra) [IFNβ1b group], and (3) Hydroxychloroquine (Single dose of 400 mg on day 1, orally, in all three arms) + Lopinavir/Ritonavir (Kaletra) (400 mg/100 mg twice a day for 10 days, orally, in all three arms) [control group]. All three groups received standards of care consisting of the necessary oxygen support, non-invasive, or invasive mechanical ventilation. Study was conducted from April 9, 2020, through April 30, 2020, at Loghman Hakim Hospital, a leading academic hospital of Shahid Beheshti University of Medical Sciences.

We tried to collect our data on a potential treatment regimen, by conducting a pragmatic randomized controlled trial for severe COVID-19 patients, without sacrificing any critical investigational component, in a reasonable time frame. Owing to the emergent nature of the study, hectic and war-like conditions in the trial site, it was not feasible to blind neither the patients nor the caregivers, but the outcomes assessor (MAP) was blinded to the study arms. Furthermore, due to the time constraints and the limited resource settings of the trial, we lacked any funding or sponsorships to prepare the required placebos.

Unstratified randomization was done in a 1:1:1 ratio utilizing a block balance randomization method. The permuted block (three or six patients per block) randomization sequence was generated using Package ‘randomizeR’ in R software version 3.6.1 and placed in individual sealed and opaque envelopes for allocation concealment by an outside statistician.

The investigator (IAD) enrolled the patients and only then opened envelopes to assign patients to the different treatment groups. This method of randomization and allocation concealment results in minimum selection and confounding biases. This trial was confirmed by the Ethics in Medical Research Committee of the Shahid Beheshti University of Medical Sciences on March 28, 2020. Signed informed consent was obtained from all of the participants or their legally authorized representatives. The trial was carried out under the Declaration of Helsinki and per the International Conference on Harmonization of Good Clinical Practice (ICH-GCP) guidelines for the conduct of clinical trials on human participants. This trial is registered with ClinicalTrials.gov, NCT04343768, and the full protocol is freely available on the BMC Trials^24^.

### Patients

Male, non-lactating, and non-pregnant female patients with at least 18 years of age who had confirmed COVID-19, defined as a positive test of Reverse Transcriptase Polymerase-Chain Reaction (RT-PCR), were screened to enter the trial. According to the medical center’s protocol, only patients with confirmed COVID-19 compatible lung involvement were admitted; as a result, all patients included in the study, also had a positive Computed Tomography Scan (CT Scan). Further eligibility criteria on admission were; [having a 1peripheral capillary oxygen saturation level (SpO_2_) ≤ 93% on pulse oximetry OR a respiratory frequency ≥ 24/minute while breathing ambient air] AND [at least one in every of the following: contactless infrared forehead thermometer temperature of ≥ 37.8, muscle ache, rhinitis, headache, cough or fatigue on admission] AND [acute onset time for the symptoms (Days ≤ 14)].

Although HCQ was administered in only a single dose, patients with cardiac arrhythmias (prolonged PR or QT intervals, third- or second-degree heart block) were excluded. Other exclusion criteria included consumption of potentially interacting medications with Lopinavir/Ritonavir + HCQ, IFNβ1a, IFNβ1b, history of alcohol use disorder, or any illicit drug dependence within the past five years, blood AST/ALT levels ≥ fivefold the maximum limit of normal range on laboratory findings and participation refusal.

### Clinical and laboratory monitoring

Vital signs (pulse rate, respiratory frequency, body temperature, and blood pressure), SpO_2_, Glasgow Coma Scale (GCS) were recorded every four hours and a seven-step ordinal scale using a protocol-defined checklist was recorded on a daily basis.

Regarding safety concerns, daily monitoring for adverse effects and laboratory testing were carried out. Nasopharyngeal swab samples were obtained before enrollment and tested using LightMix, SarbecoVIRUS E-gene RT-PCR Kits (Roche, Berlin, Germany) or Liferiver (W-RR-0479-02, China) for E, N, and Rdrp genes. Collected data were recorded on paper checklists and our Hospital Information System (HIS), which provides electronic medical records of the patients, and then double-entered into a pre-designed EXCEL sheet and later confirmed by a third investigator.

### Outcome measures

Our primary outcome measure was TTCI, defined as the time from enrollment to discharge from the hospital or a decline of two steps on the seven-step ordinal scale; whichsoever came first. Originally introduced by Beigel and colleagues in a post-hoc analysis of an influenza study as a six-step ordinal scale, and currently recommended by the WHO R&D Blueprint Team for COVID-19 studies as a nine-step ordinal scale, the utilized seven-step ordinal scale consists of the subsequent categories: (I) Not hospitalized, and has no activity limitations; (II) Not hospitalized, but has activity limitations; (III) Hospitalized, but does not need any supplemental oxygen; (IV) Hospitalized, and needs supplemental oxygen; (V) Hospitalized, and needs either High-Flow Nasal Cannula (HFNC) or non-invasive ventilation; (VI) Hospitalized, and needs invasive ventilation; and (VII) Dead^25,26^.

Secondary outcomes included mortality from the date of randomization until day 21, by which all of the patients had at least one of the following outcomes: (1) A decline of two steps on the seven-step ordinal scale, (2) Hospital discharge or (3) Death; SpO_2_ improvement defined as the difference between the last and the first recorded measurement during the hospitalization, using pulse-oximetry; length of stay in the hospital until the date of discharge from hospital or death from any cause, whichsoever came first; incidence of new mechanical ventilation use from the date of randomization until day 21. Follow-ups of discharged patients were done utilizing telemedicine visits, online, or over the telephone.

### Statistical analysis

The total sample size was calculated according to the Latouche and colleagues approach for estimating sample size in survival analysis with 80% power, alpha = 0.05, Hazard Ratio (HR) of 3.0 (as the ratio of the hazard rates of TTCI corresponding to the pooled intervention groups compared to the control group) and assuming that 80% of patients would reach the primary outcome^27^. The calculations were carried out using Package ‘powerSurvEpi’ in R and accounted for a dropout rate of 15%. With the above assumptions, 60 patients should have been recruited for this trial (20 patients in each arm).

The TTCI was determined when all the patients had reached day 21, with failure to reach the primary endpoint or death prior day 21 being regarded as right-censored.

Frequency rates and percentages were used for categorical variables, and Interquartile Ranges (IQRs) and median were used for continuous variables. Kruskal–Wallis test was used for comparing the continuous variables. The Wilcoxon signed-rank test compared the before and after intervention effects. Chi-Square test was used for comparing the frequency of categorical variables. Kaplan–Meier (compared with a log-rank test) was used to analyze the TTCI. Cox proportional-hazards model was also applied to calculate the HRs with 95% Confidence Intervals (CIs).

**Figure 1:**
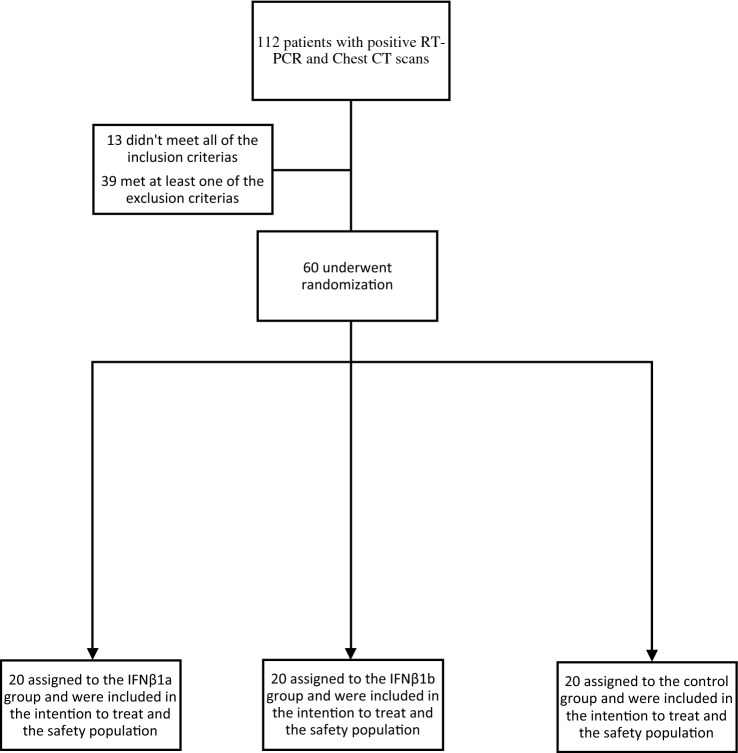
Trial flow diagram.

Intention-To-Treat (ITT) was the base population for the efficacy analysis, and all of the participants who had undergone randomization were included in it. (Fig. 1). A cutoff point of < 0.05 was used for the p-value to determine statistical significance, and all of the carried-out tests were two-tailed. R software version 3.6.1 was used to perform the statistical analyses.

## Results

### Patients

From 112 patients who were screened with positive RT-PCRs and positive Chest CT scans, 60 patients were finally enrolled in the trial; 20 were assigned to the IFNβ1a group; 20 were appointed to the IFNβ1b group, and the remaining 20 were assigned to the control group. Since all patients received the intended treatment as scheduled, the analysis just included as the ITT population (Fig. 1).

The age median for the study participants was 69 years (IQR; 55 to 82 years), and the sex distribution was almost even (51.7% men). The median period of days between the onset of symptoms and the randomization was five days (IQR, 3 to 7 days). There were no statistically significant differences, at the baseline, among the three groups regarding the demographic characteristics, clinical, or laboratory performance. (Table 1).

**Table 1:** Characteristics of the patients at baseline.

Characteristic	Total (N = 60)	Interferon Beta-1a (N = 20)	Interferon Beta-1b (N = 20)	Control (N = 20)	P-value
Age, median (IQR)—year	69.0 (55.0–82.0)	71.5 (49.7–74.8)	65.0 (57.0–74.0)	76.0 (55.0–85.0)	0.544
Male sex—no. (%)	31 (51.7%)	11 (55.0%)	9 (45.0%)	11 (55.0%)	0.766
Smoking—yes. (%)	18 (30.0%)	6 (40.0%)	4 (25.0%)	8 (44.4%)	0.478
Duration of symptoms before presentation, median (IQR)—day	5.0 (3.0–7.0)	5.5 (3.0–7.0)	6.0 (3.0–9.2)	4.0 (2.0–7.0)	0.884
Underlying conditions—no. (%)	37 (61.7%)	13 (65.0%)	12 (60.0%)	12 (60.0%)	0.932
Diabetes	14 (23.3%)	5 (25.0%)	5 (25.0%)	4 (20.0%)	0.911
Hypertension	20 (33.3%)	7 (35.0%)	6 (30.0%)	7 (35.0%)	0.928
Coronary heart disease	10 (16.7%)	5 (25.0%)	2 (10.0%)	3 (15.0%)	0.432
Chronic kidney disease	5 (8.3%)	1 (5.0%)	3 (15.0%)	1 (5.0%)	0.418
Malignancy	1 (1.7%)	0 (0.0%)	1 (5.0%)	0 (0.0%)	
Other underlying diseases	8 (13.3%)	5 (25.0%)	0 (0.0%)	3 (15.0%)	0.065
Body temperature (on admission), median (IQR)—°C	37.0 (37.0–37.0)	37.0 (37.0–37.4)	37.0 (37.0–37.0)	37.0 (36.9–37.0)	0.054
Heart rate median (IQR)	88.0 (82.0–90.0)	90.0 (87.2–93.7)	86.0 (82.0–90.0)	85.5 (76.2–93.7)	0.175
Respiratory rate median (IQR)	16.5 (16.0–20.0)	16.0 (16.0–20.0)	16.5 (15.5–19.2)	18.0 (16.0–23.0)	0.711
Respiratory Rate > 24/min—no. (%)	12 (20.7%)	3 (15.0%)	4 (22.2%)	5 (25.0%)	0.724
Systolic blood pressure < 90 mm Hg—no. (%)	0 (0.0%)	0 (0.0%)	0 (0.0%)	0 (0.0%)	1.00
Oxygen saturation (SpO_2_)—median (IQR)	87.5 (83.2–89.0)	87.0 (82.5–89.0)	85.0 (82.0–88.7)	88.0 (85.0–89.7)	0.310
Venous PaO2, median (IQR)	26.6 (20.2–39.9)	28.9 (22.6–45.3)	26.3 (20.1–34.8)	22.3 (19.4–47.9)	0.515
Venous PCO2, median (IQR)	39.9 (32.2–54.1)	36.0 (29.2–54.4)	37.8 (32.2–48.4)	47.4 (33.5–59.9)	0.229
Venous HCO3, median (IQR)	25.0 (22.2–27.0)	25.9 (21.7–27.9)	26.3 (23.7–28.4)	24.2 (21.3–26.1)	0.165
White blood cell count (× 10^−9^/L)—median (IQR)	7.1 (5.2–10.4)	6.0 (5.1–11.1)	7.2 (5.2–9.4)	7.5 (4.9–10.5)	0.879
< 4 × 10^−9^/L—no. (%)	7 (11.7%)	3 (15.0%)	3 (15.0%)	1 (5.0%)	0.723
4–10 × 10^−9^/L—no. (%)	37 (61.7%)	12 (60.0%)	13 (65.0%)	12 (60.0%)	
> 10 × 10^−9^/L—no. (%)	16 (26.7%)	5 (25.0%)	4 (20.0%)	7 (35.0%)	
Lymphocyte count (× 10^−9^/L)—median (IQR)	0.9 (0.7–1.5)	1.0 (1.0–1.5)	0.9 (0.8–1.4)	0.8 (0.7–1.7)	0.959
≥ 1.0 × 10^−9^/L—no. (%)	24 (40.0%)	10 (50.0%)	7 (35.0)	7 (35.0%)	0.535
< 1.0 × 10^−9^/L—no. (%)	35 (60.0%)	10 (50.0%)	13 (65.0%)	13 (65.0%)	
Neutrophil count (× 10^−9^/L)—median (IQR)	5.1 (3.4–8.8)	4.9 (3.4–9.0)	5.4 (3.3–7.5)	5.1 (3.5–9.1)	0.966
≥ 1.5 × 10^−9^/L—no. (%)	3 (5.0%)	1 (5.0%)	2 (10.0%)	0 (0.0%)	0.641
1.5–8 × 10^−9^/L—no. (%)	40 (66.7%)	14 (70.0%)	13 (65.0%)	13 (65.0%)	
> 8 × 10^−9^/L—no. (%)	17 (28.3%)	5 (25.0%)	5 (25.0%)	7 (35.0%)	
Platelet count (× 10^−9^/L)—median (IQR)	200.0 (159.2–247.7)	194.5 (159.2–250.0)	212.5 (168.2–249.5)	188.5 (154.5–242.0)	0.707
≥ 100 × 10^−9^/L—no. (%)	59 (98.3%)	20 (100.0%)	20 (100.0%)	19 (95.0%)	0.362
< 100 × 10^−9^/L—no. (%)	1 (1.7%)	0 (0.0%)	0 (0.0%)	1 (5.0%)	
Serum creatinine (μmol/L)—median (IQR)	97.2 (88.4–130.4)	97.2 (88.4–119.3)	97.2 (88.4–114.9)	114.9 (81.8–165.7)	0.564
≤ 133 μmol/L—no. (%)	49 (81.7%)	17 (85.0%)	17 (85.0%)	15 (75.0%)	0.641
> 133 μmol/L—no. (%)	11 (18.3%)	3 (15.0%)	3 (15.0%)	5 (25.0%)	
Aspartate aminotransferase (AST) (U/L)—median (IQR)	50.0 (32.0–78.0)	45.0 (30.0–87.0)	40.5 (32.7–53.5)	62.0 (39.5–77.5)	0.203
≤ 40 U/L—no. (%)	24 (41.4%)	10 (50.0%)	9 (50.0%)	5 (25.0%)	0.185
> 40 U/L—no. (%)	34 (58.6%)	10 (50.0%)	9 (50.0%)	15 (75.0%)	
Alanine Aminotransferase (ALT) (U/L)—median (IQR)	38.0 (28.0–51.0)	44.5 (26.2–66.7)	31.0 (27.7–40.0)	40.0 (31.2–53.7)	0.119
≤ 50 U/L—no. (%)	43 (74.1%)	13 (65.0%)	17 (94.4%)	13 (65.0%)	0.06
> 50 U/L—no. (%)	15 (25.9%)	7 (35.0%)	1 (5.6%)	7 (35.0%)	
Lactate Dehydrogenase (LDH) (U/L)—median (IQR)	536.0 (339.0–776.5)	550.0 (328.0–771.0)	470.5 (327.7–638.5)	598.0 (431.0–1100.0)	0.427
≤ 245 U/L—no. (%)	2 (5.6%)	1 (7.7%)	1 (8.3%)	0 (0.0%)	0.626
> 245 U/L—no. (%)	34 (94.4%)	12 (92.3%)	11 (91.7%)	11 (100.0%)	
Blood urea nitrogen (BUN)—median (IQR)	42.0 (29.0–59.0)	40.0 (25.0–55.0)	45.0 (32.7–60.5)	41.5 (29.0–98.7)	0.505
C-Reactive Protein (CRP)—median (IQR)	57.6 (14.0–78.5)	52.0 (19.3–83.8)	46.6 (13.2–78.5)	70.3 (14.1–78.2)	0.877
CRP < 6—no. (%)	3 (7.1%)	2 (13.3%)	1 (8.3%)	0 (0.0%)	0.359
CRP > 6—no. (%)	39 (92.9%)	13 (86.7%)	11 (91.7%)	15 (100.0%)	
Erythrocyte sedimentation rate (ESR)—median (IQR)	45.0 (14.7–62.5)	43.5 (14.7–50.7)	64.0 (40.0–74.0)	31.0 (9.0–50.0)	0.030

The values shown are based on available data. Laboratory values for Aspartate Aminotransferase and Alanine Aminotransferase were available for 18 patients in the Interferon Beta-1b group. Values for Lactate Dehydrogenase were available for 13 patients in Interferon Beta-1a, 12 patients in the Interferon Beta-1b, and 11 patients in the control group. Values for C-Reactive Protein were available for 15 patients in Interferon Beta-1a, 12 patients in the Interferon Beta-1b, and 15 patients in the control group. IQR denotes the interquartile range. Quantitative measures were compared using the Kruskal–Wallis test. Categorical variables were compared using the Chi-Square test.

### Primary outcome

**Figure 2:**
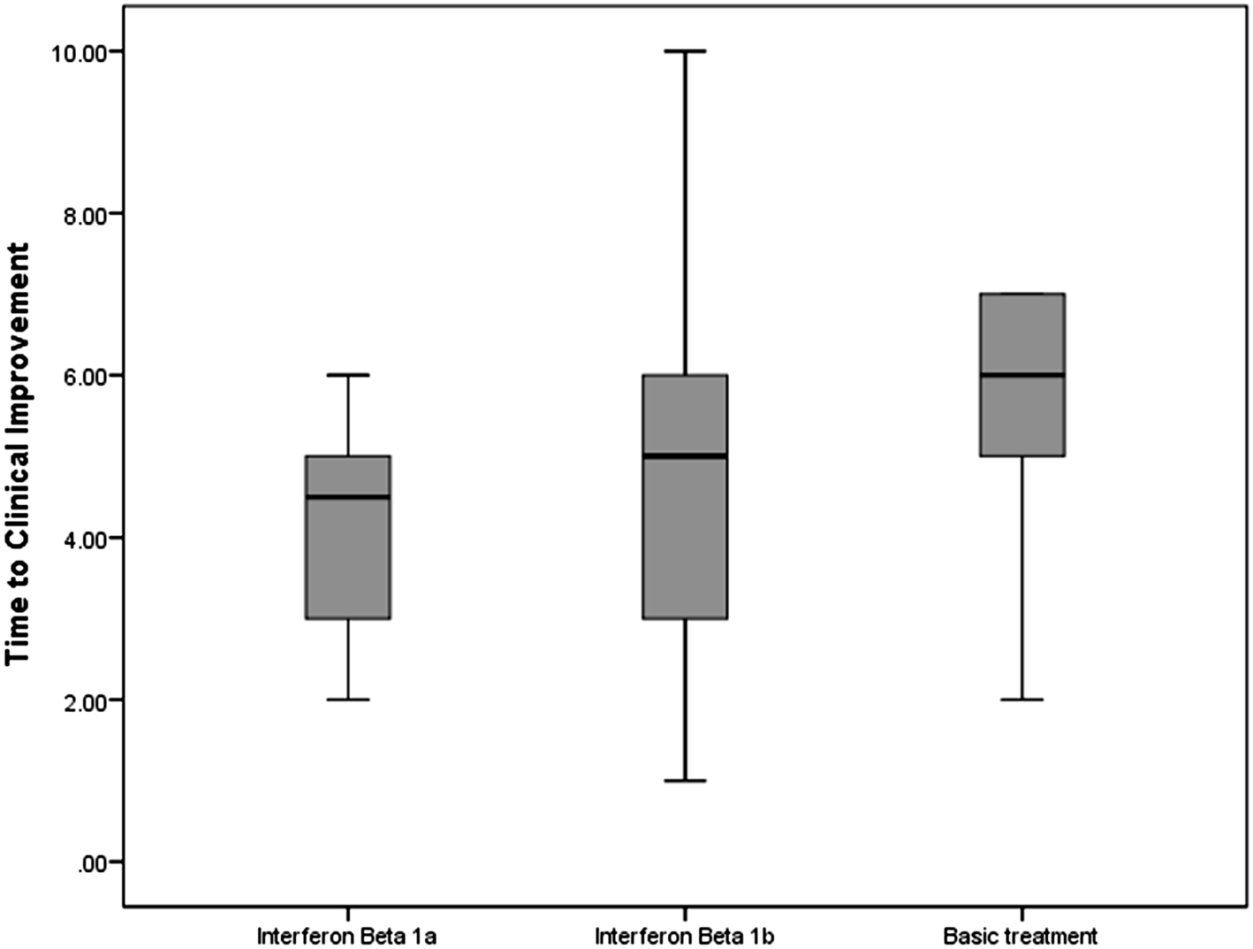
Box-plots of time to clinical improvement across three treatment groups for those patients who reached the improvement (The patients who experienced mortality were omitted from this box-plots).

**Figure 3:**
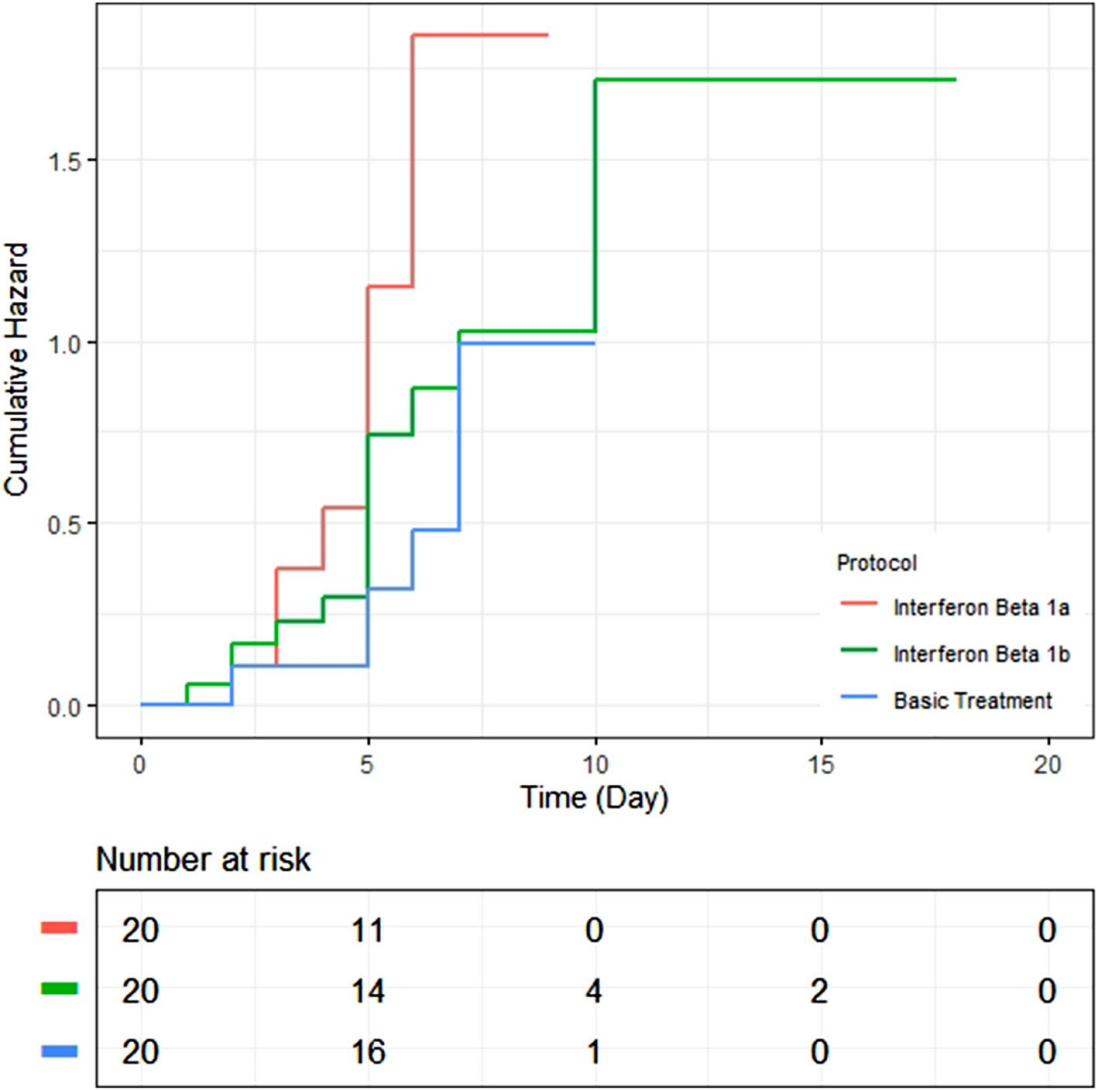
Time to clinical improvement in the intention-to-treat population.

Patients appointed to the IFN groups showed a TTCI different from the control group in the ITT population (median, five days for both of the intervention groups vs. seven days for the control group; P-value = 0.046) (Table 2) (Figs. 2, 3). According to 95%CI, the TTCI for IFNβ1a group was significantly lower than the control group, while the IFNβ1b group was not significantly different from the controls.

**Table 2:** Outcomes in the intention-to-treat population.

Characteristic		Total (N = 60)		Interferon Beta-1a (N = 20)	Interferon Beta-1b (N = 20)	Control (N = 20)		P-value
Time to clinical improvement—median (95% CI)		6.0 (5.2–6.7)		5.0 (4.2–5.7)	5.0 (3.6–6.4)	7.0 (6.1–7.9)		0.046
Mortality at day 21—no. (%)		19 (31.7%)		4 (20.0%)	6 (30.0%)	9 (45.0%)		0.231
Mortality in early presentation (≤ 6 days of symptom onset)—no. (%)		9 (26.5%)		2 (16.7%)	3 (30.0%)	4 (33.3%)		0.623
Mortality in late presentation (> 6 days of symptom onset)—no. (%)		10 (38.5%)		2 (25.0%)	3 (30.0%)	5 (62.5%)		0.238
ICU admission—no. (%)		45 (75.0%)		16 (80.0%)	13 (65.0%)	16 (80.0%)		0.449
Invasive mechanical ventilation—no. (%)		21 (35.0%)		7 (35.0%)	7 (35.0%)	7 (35.0%)		1.00
Hospital stay—median no. of days (IQR)		5.0 (3.2–7.0)		5.0 (3.0–6.0)	5.0 (3.2–7.7)	6.0 (5.0–7.0)		0.312
Time from enrollment to discharge—median no. of days (IQR)		5.0 (3.0–6.0)		4.0 (3.0–5.0)	5.0 (2.7–6.2)	5.5 (5.0–7.0)		0.183
Time from enrollment to death—median no. of days (IQR)		7.0 (5.0–9.0)		8.0 (4.0–9.0)	7.5 (4.2–18.0)	7.0 (4.5–8.0)		0.813
Mean body temperature (during hospitalization), median (IQR)—°C		36.9 (36.8–37.1)		36.9 (36.9–37.1)	36.9 (36.7–37.1)	36.8 (36.7–37.0)		0.227
Mean respiratory rate median (IQR)		16.5 (14.5–18.7)		15.8 (14.0–18.4)	16.5 (14.5–22.2)	16.8 (15.5–18.7)		0.416
Last respiratory rate median (IQR)		16.0 (14.0–18.0)^†^		16.0 (14.0–18.0)^†^	16.0 (14.0–24.7)	16.0 (14.2–18.0)^†^		0.610
Mean oxygen saturation (SpO_2_)—median (IQR)		88.4 (86.1–90.1)		89.0 (86.0–91.0)	87.7 (83.1–89.2)	88.5 (86.2–91.0)		0.326
Worst oxygen saturation (SpO_2_)—median (IQR)		85.0 (82.0–89.0)		86.0 (82.0–89.0)	83.0 (80.0–88.0)	85.5 (84.2–89.0)		0.352
Last oxygen saturation (SpO_2_)—median (IQR)		90.0 (88.0–94.0)^†^		90.5 (89.0–95.7)^†^	90.0 (84.0–93.0)	90.0 (85.0–93.2)^†^		0.410
White blood cell count (× 10^−9^/L)—median (IQR)		7.2 (6.1–10.4)		7.2 (5.2–9.9)	7.1 (6.3–9.7)	9.2 (6.5–12.2)		0.450
< 4 × 10^−9^/L—no. (%)		2 (4.0%)		2 (11.1%)	0 (0.0%)	0 (0.0%)		0.223
4–10 × 10^−9^/L—no. (%)		34 (68.0%)		12 (66.7%)	12 (80.0%)	10 (58.8%)		
> 10 × 10^−9^/L—no. (%)		14 (28.0%)		4 (22.2%)	3 (20.0%)	7 (41.2%)		
Lymphocyte count (× 10 − 9/L)—median (IQR)		1.03 (0.63–1.40)		1.12 (0.69–1.40)	1.04 (0.64–1.70)	0.81 (0.62–1.25)		0.304
≥ 1.0 × 10^−9^/L—no. (%)		25 (51.0%)		12 (66.7%)	8 (53.3%)	6 (35.3%)		0.177
< 1.0 × 10^−9^/L—no. (%)		24 (49.0%)		6 (33.3%)	7 (46.7%)	11 (64.7%)		
Platelet count (× 10^−9^/L)—median (IQR)		197.0 (160.7–268.5)		191.0 (159.0–272.0)	219.5 (152.5–262.2)	193.0 (169.5–292.0)		0.785
≥ 100 × 10^−9^/L—no. (%)		51 (98.1%)		19 (100.0%)	16 (100.0%)	16 (94.1%)		0.350
< 100 × 10^−9^/L—no. (%)		1 (1.9%)		0 (0.0%)	0 (0.0%)	1 (5.9%)		
Neutrophil count (× 10^−9^/L)—median (IQR)		5.9 (4.6–5.9)		5.3 (3.8–8.6)	5.3 (4.3–6.5)	8.3 (6.0–11.1)		0.092
≥ 1.5 × 10^−9^/L—no. (%)		1 (2.2%)		1 (5.6%)	0 (0.0%)	0 (0.0%)		0.128
1.5–8 × 10^−9^/L—no. (%)		29 (64.4%)		12 (66.7%)	11 (84.6%)	6 (42.9%)		
> 8 × 10^−9^/L—no. (%)		15 (33.3%)		5 (27.8%)	2 (15.4%)	15 (57.1%)		
Serum creatinine (μmol/L)—median (IQR)		88.4 (79.6–154.7)		88.4 (75.1–106.1)	79.5 (79.5–106.1)	106.1 (79.5–185.6)		0.549
≤ 133 μmol/L—no. (%)		44 (73.3%)		17 (85.0%)	16 (80.0%)	11 (55.0%)		0.071
> 133 μmol/L—no. (%)		16 (26.7%)		3 (15.0%)	4 (20.0%)	9 (45.0%)		
Last BUN—median (IQR)		50.0 (34.0–102.2)		38.0 (31.0–90.5)	36.0 (30.0–74.7)	80.0 (39.0–117.0)		0.107

The values are based on analysis of available data. Laboratory values for White Blood Cell count and Lymphocyte count were available for 18 patients in Interferon Beta-1a, 15 patients in the Interferon Beta-1b, and 17 patients in the control group. Values for Platelet count were available for 19 patients in Interferon Beta-1a, 16 patients in the Interferon Beta-1b, and 17 patients in the control group. Values for Neutrophil count were available for 13 patients in Interferon Beta-1a, 11 patients in the Interferon Beta-1b, and 6 patients in the control group. IQR denotes the interquartile range. Quantitative measures were compared using the Kruskal–Wallis test. Categorical variables were compared using the Chi-Square test. Comparisons before and after intervention have been made using The Wilcoxon signed-rank test.

^†^Statistically significant in comparison to the baseline.

Also, HRs for TTCI in the Cox regression model were 2.36; (95% CI 1.10–5.17, P-value = 0.031) for IFNβ1a group, and 1.42, (95% CI 0.63–3.16, P-value = 0.395) for the IFNβ1b group.

### Secondary outcomes

A total of 19 patients died during the study. The in-hospital mortality was numerically lower in both of the intervention groups than the control group in both ITT population (20% in the group of IFNβ1a and 30% in the group of IFNβ1b vs. 45% in the control group) (Table 2), but the observed differences did not reach the edge of statistical significance, probably due to the underpowered nature of our study.

There was no significant decrease in the incidence of the need for invasive mechanical ventilation among the three groups. All other secondary outcome measures, although showing numerically favorable values in both of the interferon arms, did not reach statistical significance, both when compared between the two intervention groups and when compared with the control group. Furthermore, the last SpO_2_ was statistically higher than its baseline values for both of the treatment groups, but not for the control group (Table 2).

### Safety

We enrolled only severe patients, and unfortunately, the rates of many of the adverse events were high in all three groups. The most common adverse event faced during the trial was abnormalities in the biomarkers of liver injury, and the most common severe adverse effect was Acute Respiratory Distress Syndrome (ARDS). Even though there were no significant differences between the three arms regarding the safety aspect, the control group had the worst overall adverse events profile, numerically (Table 3). No patient stopped treatment because of the adverse events.

**Table 3:** Adverse events in the safety population.

Event	Interferon Beta-1a (N = 20)	Interferon Beta-1b (N = 20)	Control (N = 20)
**Adverse event**			
Nausea	4 (20%)	5 (25%)	5 (25%)
Vomiting	1 (5%)	0 (0%)	1 (5%)
Diarrhea	3 (15%)	4 (20%)	3 (15%)
Rash	0 (0%)	0 (0%)	1 (5%)
Increased alanine aminotransferase (ALT)	9 (45%)	6 (30%)	8 (40%)
Increased aspartate aminotransferase (AST)	12 (60%)	13 (65%)	16 (80%)
Hyperbilirubinaemia	2 (10%)	1 (5%)	1 (5%)
Increased creatinine	4 (20%)	3 (15%)	6 (30%)
Prolonged QT interval	0 (0%)	0 (0%)	1 (5%)
**Serious adverse event**			
Acute respiratory distress syndrome (ARDS)	7 (35%)	8 (40%)	10 (50%)
Acute kidney failure (AKI)	5 (25%)	4 (20%)	6 (30%)
Shock	1 (5%)	1 (5%)	1 (5%)

Adverse events that occurred in more than one patient after randomization through day 21 are shown. Some patients had more than one adverse event. All deaths were due to respiratory failure.

No statistically significant differences were observed between the three groups.

## Discussion

In summary, the COVIFERON trial unveiled that both IFN intervention groups had numerically more favorable TTCIs when compared to the control group; however, the numbers were statistically significant only for IFNβ1a. Furthermore, the mortality rate in the control group was more than two-fold higher than that of the IFNβ1a group, although not reaching the verge of statistical significance, which might be explained on the basis of our reduced study power.

Although the clinical efficacy of Lopinavir/Ritonavir and HCQ is under genuine scrutiny, it was impossible for us not to include this regimen in all three arms, as this combination was mandated for all severely ill COVID-19 patients by the Iranian COVID-19 national protocol, endorsed by the Iranian Ministry of Health^10–13,28,29^.

Innate and adaptive immunity are the two fundamental arms of the immune system in vertebrates. During microbial infections, innate immunity is the first arm that comes into action. In the course of a viral infection, following primary implantation, local replication, and spread of the virus, to susceptible neighboring host cells, the innate immune system acts first and initiates several downstream cellular signaling cascades that lead to the formation of pro-inflammatory cytokines and IFNs promoting a prompt immune response. IFNs stimulate the expression of several genes that contribute to shifting the host cells toward an antiviral state, hindering dissemination, and secondary replication of the virus. Although three major types of IFNs, type I, II, and III, have been identified, type I IFN has a crucial function in the antiviral response and modulates the ensuing adaptive immune responses. Accordingly, decreased expression of type I IFNs and/or IFN induced genes, hinder prompt innate, and adaptive immune responses. Indeed, the immediate and integrated innate and consequent adaptive immune response is the mainstay of the antiviral defense mechanism. An attenuated initial innate immune response could be followed by dysregulated, excessive, and destructive adaptive immune reactions. SARS-CoV and MERS-CoV suppress the expression of type I IFNs and IFN induced genes, thereby disrupt both the innate and the adaptive responses of the immune system, which contribute to the tenacious pathogenesis of the virus. Henceforth and with the significant similarities observed between COVID-19 and the two previous diseases, regarding the changes in the total neutrophil and lymphocytes counts in patients, it is heavily postulated that SARS-CoV-2 may also inhibit the type I IFNs in the early phase of COVID-19 disease. The resultant dysregulated innate immune responses could, in turn, escalate to further dissemination and secondary replication of the virus, followed by an excessive and destructive subsequent adaptive immune response^16,17,30–34^. Accordantly, we found a meaningful correlation between the administration of IFNβ1a and the alleviation of the clinical course of COVID-19 disease. This observation was of particular importance, since it could propose IFN as a potent therapeutic option in severe COVID-19 cases.

Channappanavar and colleagues recommend that the usage of IFNs should be limited to the initial stages of infection because of safety concerns over its pro-inflammatory side-effects if administered at later stages; furthermore, the effects are presumably stronger with earlier initiation^31^. Although we did not observe any statistically significant adverse effects related to the later administration of IFN, we firmly believe that more robust effects could have been obtained, had the time between the first symptom and the first dose of IFN been minimized.

Recently Hung and colleagues presented their results on the triple therapy combination of IFNβ1b + Ribavirin + Lopinavir/Ritonavir compared against Lopinavir/Ritonavir monotherapy. They enrolled only mild to moderate cases and revealed that the combination group had a much faster clinical recovery and a narrower viral shedding window, which could imply less infectivity. Post-hoc analysis also indicated more substantial effects with an earlier initiation (less than seven days after the symptom onset) of the combination therapy. Their interesting data analysis demonstrated an impressive HR of 4.37 (95% CI 1.86–10.24) for the primary outcome measure of time to a negative nasopharyngeal swab. Even though a subgroup comparison, with a small number of patients, suggested that IFNβ1b was a crucial component of the combination regimen, the observed HR cannot be pinned to any of the single treatments used in combination^35^. With the higher HRs for the TTCI observed in our study in the IFNβ1a group compared to the IFNβ1b group, we can argue that the future trials should be focused on both types of the IFNs; even with a bigger emphasis on IFNβ1a.

To the best of our knowledge, our trial is the first randomized, controlled trial to evaluate the possible efficacy and safety of exogenously administered IFNβ1a and IFNβ1b on the course and outcomes of hospitalized patients with severe COVID-19 disease. Furthermore, our exhaustive literature search through the PubMed, EMBASE, Web of Science, Google Scholar, Scopus, CINAHL, Ovid, Cochrane CENTRAL, ClinicalTrials.gov, and WHO’s International Clinical Trials Registry Platform (ICTRP) databases for published, ongoing or future trials indicated that unfortunately, our study is the only RCT designed to assess the different types of interferons in the same study against a control group for this disease. With the current promising results delineated by our trial, better-designed, larger, multicenter, and adequately powered studies are needed to confirm or refute our findings.

Our study has several limitations. The trial was open-label and without a placebo-control group, which opens the possibility for risks of bias. Our study was underpowered due to the reduced realized OR compared to the initial presumed OR, hence generalizing the findings of our trial regarding the IFNβ1b should be exercised with caution. Our trial was carried out in a limited resource setting, where we had no access to the follow-up RT-PCT testing and quantitative Real-Time RT-PCR; therefore, we were unable to determine the time to a negative RT-PCR test and the viral loads to shed further light on the effect of the studied drugs on viral dynamics. As discussed before, earlier administration of exogenous IFNs might have yielded more substantial results, but the meantime, from the symptom onset to the first dose of IFN in our study was 5.4 days. Finally, we only enrolled the severe patients with lower probabilities of survival; hence our findings cannot be extrapolated to all COVID-19 patients. Interestingly lack of funding and the absence of any potential conflicts of interest could be accounted as a strength for this study.

In conclusion, we showed that IFNβ1a, seems to be a reasonable choice for severe COVID-19 patients, due to the excellent safety profile and possible benefits. We showed statistically significant reductions in TTCI for IFNβ1a but only numerical reductions for IFNβ1b; which might be due to the underpowered nature of our study. We furthermore found that IFNβ1a was superior when compared to IFNβ1b regarding the primary outcome. Additional studies are urgently warranted to further elaborate on the importance of IFNs in tackling the current COVID-19 pandemic.

## Supplementary Information

Supplementary Information 1.

Supplementary Information 2.

Supplementary Information 3.

## Data Availability

Qualified researchers can submit proposals to the corresponding author with a valuable research question (with relevant approvals including ethical approval) to request access to any of the deidentified datasets of this clinical trial. A formal contract will be signed and an independent data protection agency should oversee the sharing process to ensure the safety of the participants data.
