# Supplementary material for: Role of interferon therapy in severe COVID-19: the COVIFERON randomized controlled trial
Source: Sci Rep. 2021 Apr 13;11:8059. doi: 10.1038/s41598-021-86859-y (PMC8044200; doi:10.1038/s41598-021-86859-y)
Supplement: Supplementary file 2 — Supplementary Information 2. [file 41598_2021_86859_MOESM2_ESM.docx]

**Role of Interferon Therapy in Severe COVID-19: The COVIFERON Randomized Controlled Trial**

Ilad Alavi Darazam, MD ^1,2^, Prof. Shervin Shokouhi, MD ^1,2^, Mohamad Amin Pourhoseingholi, PhD ^3^, Seyed Sina Naghibi Irvani, MD, MPH, MBA ^1*^, Majid Mokhtari, MD ^4^, Minoosh Shabani, MD^1,2^, Mahdi Amirdosara, MD ^5^, Parham Torabinavid, MD ^6^, Maryam Golmohammadi, MD ^1^, SayedPayam Hashemi, MD ^1^, Arsalan Azimi, MD ^7^, Mohammad Hossein Jafarazadeh Maivan, MD ^8^, Prof. Omidvar Rezaei, MD ^9^, Prof. Alireza Zali, MD ^10^, Mohammadreza Hajiesmaeili, MD ^5^, Hadiseh Shabanpour Dehbsneh, MD ^1,2^, Akram Hoseyni Kusha, MD ^1,2^, Maryam Taleb Shoushtari, MD ^1,2^, Negar Khalili, MD ^1,2^, Azam Soleymaninia, MD ^1,2^, Prof. Latif Gachkar, MD ^1,2^, Ali Khoshkar, MD ^11^. [SBMU Taskforce on The COVIFERON Study]

**^1^** Department of Infectious Diseases, Loghman Hakim Hospital, Shahid Beheshti University of Medical Sciences, Tehran, Iran.

**^2^** Infectious Diseases and Tropical Medicine Research Center, Shahid Beheshti University of Medical Sciences, Tehran, Iran.

**^3^** Basic and Molecular Epidemiology of Gastrointestinal Disorders Research Center, Research Institute for Gastroenterology and Liver Diseases, Shahid Beheshti University of Medical Sciences, Tehran, Iran.

**^4^** Department of Pulmonary and Critical Care Medicine, Loghman Hakim Hospital, Shahid Beheshti University of Medical Sciences, Tehran, Iran.

**^5^** Anesthesiology Research Center Loghman Hakim Hospital, Shahid Beheshti University of Medical Sciences, Tehran, Iran.

**^6^** Student Research Committee, Hamadan University of Medical Sciences, Hamadan, Iran.

**^7^** Shiraz University of medical sciences, Zand Blvd., Shiraz, Iran.

**^8^** Clinical Research Development Unit, Bohlool Hospital, School of Medicine, Gonabad University of Medical Sciences, Gonabad, Iran.

**^9^** Skull Base Research Center, Loghman Hakim Hospital, Shahid Beheshti University of Medical Sciences, Tehran, Iran.

**^10^** Functional Neurosurgery Research Center, Shohada Tajrish Neurosurgical Center of Excellence, Shohada Tajrish Hospital, Shahid Beheshti University of Medical Sciences, Tehran, Iran.

**^11^** Department of Surgery, Loghman Hakim Hospital, Shahid Beheshti University of Medical Sciences, Tehran, Iran.

**^*^Corresponding Author:**

Seyed Sina Naghibi Irvani, MD, MPH, MBA,
Department of Infectious Diseases, Loghman Hakim Hospital, Shahid Beheshti University of Medical Sciences, Tehran, Iran. (**Email:** Sina.Irvani@sbmu.ac.ir)
**P.O. Box:** 1567812907, **Tel:** +98-914-1182825, **Fax:** +98-413-3845238

**Protocol**

This trial protocol has been provided by the authors to give readers additional information about their work.

**Effectiveness of Interferon Beta 1a and Interferon Beta 1b compared to the Usual Therapeutic Regimen to Treat Adults with Moderate to Severe COVID-19**

A single center, open label, randomized, controlled, parallel group, clinical trial

**Clinical Study Protocol**

**Trial Code:** NCT04343768

**Sponsored by:** Shahid Beheshti University of Medical Sciences

**Study Phase:** Phase II

**Indication for Present Study:** The current outbreak of COVID19 and difficulties caused by the COVID-19 pandemic

**Trial Protocol Authors:**

Dr. Seyed Sina Naghibi Irvani, MD, MPH, MBA

Dr. Maryam Golmohammadi, MD

Dr. Mohamad Amin Pourhoseingholi, PhD

Dr. Ilad Alavi Darazam, MD

Dr. Shervin Shokouhi, MD

**Coordinating Center:** Loghman Hakim Hospital

**Table of Contents**

[**PROTOCOL SUMMARY** 4](#_Toc42265555)

[**INTRODUCTION** 7](#_Toc42265556)

[**Background** 7](#_Toc42265557)

[**STUDY OBJECTIVES** 9](#_Toc42265558)

[**Primary objective** 9](#_Toc42265559)

[**Secondary objectives** 9](#_Toc42265560)

[**Practical Objective** 10](#_Toc42265562)

[**Hypothesis** 10](#_Toc42265563)

[**STUDY DESIGN** 11](#_Toc42265564)

[**General** 11](#_Toc42265565)

[**Safety Assessments** 12](#_Toc42265566)

[**Efficacy Assessments** 12](#_Toc42265567)

[**Study Outcomes** 12](#_Toc42265568)

[**Study Drugs** 13](#_Toc42265569)

[**Statistical Methods** 14](#_Toc42265570)

[**STUDY POPULATION** 15](#_Toc42265571)

[**Participants** 15](#_Toc42265572)

[**Inclusion Criteria** 15](#_Toc42265573)

[**Exclusion Criteria** 16](#_Toc42265574)

[**Drop Out** 16](#_Toc42265575)

[**RESTRICTION** 16](#_Toc42265576)

[**ETHICAL CONCIDERATION** 17](#_Toc42265577)

[**REFERENCES** 17](#_Toc42265578)

# **PROTOCOL SUMMARY**

**Study Title:**

Effectiveness of Interferon Beta 1a and Interferon Beta 1b compared to the Usual Therapeutic Regimen to Treat Adults with Moderate to Severe COVID-19

**Objectives:**

We will investigate the effectiveness of Interferon Beta 1a, compared to Interferon Beta 1b and the usual therapeutic regimen in COVID-19 in patients that have tested positive and are moderately to severely ill.

**Trial Design:**

This is a single center, open label, randomized, controlled, parallel group, clinical trial that will be conducted at Loghman Hakim hospital in conjunction with Shahid Beheshti University of Medical Sciences.

**Participants:**

Sixty COVID-19 confirmed cases (using the RT-PCR test) will be enrolled in the trial between April 9^th^ to April 14^th^ 2020. Patients will be randomly assigned to the intervention groups or the control group with the following eligibility criteria: ≥ 18 years of age AND (oxygen saturation (SPO2) ≤ 93% OR respiratory rate ≥ 24) AND at least one of the following: Contactless infrared forehead thermometer temperature of ≥37.8°C, cough, sore throat, nasal congestion/drip, myalgia, headache or fatigue on admission, and onset of the symptoms should be acute (Days ≤ 14). Although Hydroxychloroquine will be administered in a single dose, patients with heart problems (prolonged corrected QT (450 milliseconds) or PR intervals, second- or third-degree heart block, and history of arrhythmias including torsade de pointes) will be excluded. Other exclusion criteria include using drugs with potential interaction with Hydroxychloroquine + Lopinavir/Ritonavir, Interferon-β 1a, Interferon-β 1b, pregnant or lactating women, history of alcohol or drug addiction in the past 5 years, blood ALT/AST levels > 5 times the upper limit of normal on laboratory results and refusal to participate.

This study will be undertaken at the Loghman Hakim Hospital, Shahid Beheshti University of Medical Sciences.

**Intervention and Comparator:**

COVID-19 confirmed patients will be randomly assigned to one of three groups, with 20 patients in each. The first group (Arm 1) will receive Hydroxychloroquine + Lopinavir / Ritonavir (Kaletra) + Interferon-β 1a (Recigen), the second group (Arm 2) will be administered Hydroxychloroquine + Lopinavir / Ritonavir (Kaletra) + Interferon-β 1b (Ziferon), and the control group (Arm 3) will be treated by Hydroxychloroquine + Lopinavir / Ritonavir (Kaletra).

**Efficacy Assessments:**

**Primary outcome:**

Time to clinical improvement is our primary outcome measure. This is an improvement of two points on a seven-category ordinal scale (recommended by the World Health Organization: Coronavirus disease (COVID-2019) R&D. Geneva: World Health Organization) or discharge from the hospital, whichever comes first.

**Secondary outcomes:**

Secondary outcomes include mortality from the date of randomization until the last day of the study which will be the day all of the patients have had at least one of the following outcomes: 1) Improvement of two points on a seven-category ordinal scale. 2) Discharge from the hospital 3) Death. If any patient dies, we have reached an important secondary outcome. SpO2 Improvement between the last and first day of hospitalization, using pulse-oximetry. Duration of hospitalization from date of randomization until the date of hospital discharge or date of death from any cause, whichever comes first. Incidence of new mechanical ventilation uses from date of randomization until the last day of the study. Please note that we are trying to add further secondary outcomes and this section of the protocol is still evolving.

**Statistical Methods:**

Statistical analysis will be performed by R version 3.6.1 software. We will use Kaplan–Meier to analyze the time to clinical improvement (compared with a log-rank test). Hazard ratios with 95% confidence intervals will be calculated using the Cox proportional-hazards model in crude and adjusted analysis.

# **INTRODUCTION**

**Background**

As of 1^th^ March, 2020, a cumulative total of 8,116 Coronavirus (COVID-19) cases were detected in China and 11,011 patients out of China. Among Chinese patients, the death was 3,231, or 3.98%. While the number of deaths due to COVID-19, outside China has been reported as 4576 cases. So far in Iran, the COVID19 test of 16169 people has been reported positively and the number of deaths has been 988 individuals, with 6.11% mortality rates (1).

Coronavirus SARS-CoV-2, an enveloped RNA virus, is responsible for severe respiratory illness and viral pneumonia around the world. As COVID-19 is a new pathogen, everyone is assumed to be susceptible and there is no known pre-existing immunity in humans. So, according to epidemiologic reports from china and other countries, it has become a global health emergency (2).

SARS-CoV-2 belongs to the β-coronavirus cluster, as do SARS-CoV and MERS-CoV (3). However, it is not SARS or influenza, it is a novel virus with its own features. Generally, Viral infection in mammalian cells activates the body's innate immune system and interferons (IFNs) play a key role in this event because they activate the innate immune system and also contribute to acquired immunity. There are two types of IFN involved in antiviral activity: Type I is synthesized by most cell types as a direct response to the virus, while type II is produced by immune cells after contact with cells containing antigens. Both type I and type II have antiviral, anti-proliferative, and immune system modulating properties (4). In animal model with SARS-CoV infection, IFN imbalance of type I and inflammatory cells was shown to be the leading cause of fatal pneumonia (5). According to lack of information available on COVID-19, SARS could be a useful model in this regard. Because SARS-CoV-2 resemble to SARS-CoV in structure and nucleotide sequence among other viruses of this family (6), another study found that Coronavirus targets the most important proteins in the IFN signaling pathway to fight the immune system. This study highlighted the main role of the IFN-mediated antivirus in responding to Coronavirus infection (7).

IFN-β 1a is currently used for a variety of diseases, including multiple sclerosis, and has been shown to be effective in several dose-dependent clinical trials. Significantly, IFN-β 1a has a strong antiviral activity have shown in previous studies and also has an acceptable safety profile. In a 2003 study, SARS was treated with human interferons and found that IFN-β was 5 to 10 times more effective than other types of interferons and was the best antiviral drug against SARS-CoV (8).

Due to COVID-19 outbreak, the novel nature, astonishing speed of spreading, severe disease, death and limited data, further studies are required to fill this knowledge gap. So we also aim to examine the therapeutic effect of Interferon Beta 1a, compared to Interferon Beta 1b and the usual therapeutic regimen in COVID-19 in patients that have tested positive and are moderately to severely ill.

Since the recent global outbreak of COVID19, extensive attempts were performed to better understand the new virus and its clinical features. Although lots of clinical trials were conducted, key knowledge gaps remain and so far there is not definitive and effective treatment to manage the disease.

Our study introduces a new therapeutic regimen that reduces mortality rates, length of hospital stay (LOHS) and improves clinical symptoms in patients with COVID-19. So the present therapeutic regimen, as a promising solution, could help health care systems to deal with difficulties caused by the COVID-19 pandemic.

Moreover, to the best of our knowledge, this is the first report, investigating the effectiveness of Interferon Beta 1a, compared to Interferon Beta 1b and the usual therapeutic regimen in COVID-19 confirmed cases.

# **STUDY OBJECTIVES**

**Primary objective**

We will investigate the effectiveness of Interferon Beta 1a, compared to Interferon Beta 1b and the usual therapeutic regimen in COVID-19 in patients that have tested positive and are moderately to severely ill.

**Secondary objectives**

The secondary objectives for this study are:

1. To determine and compare the frequency of side effects in three therapeutic arms
2. To determine and compare the average level of oxygen saturation (Sat O2) in three therapeutic arms.
3. To determine and compare the average respiratory rate (RR) in three therapeutic arms
4. To determine and compare the average length of hospital stay in three therapeutic arms
5. To determine and compare the average length of hospital stay in the intensive care unit (ICU) between three therapeutic arms
6. To determine and compare the frequency of 30-days mortality rates between three therapeutic arms
7. To determine and compare the average time to improve clinical symptoms between three therapeutic arms
8. To determine and compare the average SOFA score in three therapeutic arms
9. To determine and compare the average duration of mechanical ventilation use between three therapeutic arms

**Practical Objective**

To introduce a new therapeutic regimen in order to reduce mortality rates and improve related symptoms in COVID-19 patients.

**Hypothesis**

- The frequency of side effects varies in each of the 3 treatment groups
- The average level of oxygen saturation (Sat O2) varies in each of the 3 treatment groups
- The average respiratory rate (RR) in each of the 3 treatment groups is different
- The average length of hospital stay varies in each of the 3 treatment groups
- The average length of hospital stay in ICU varies in each of the 3 treatment groups
- The frequency of 30-day mortality varies in each of the 3 treatment groups
- The average time until the nasopharyngeal swap test is negative in each of the 3 treatment groups is different
- The average time to clinical improvement in each of the 3 treatment groups is different
- The average SOFA score varies in each of the 3 treatment groups
- The average duration of mechanical ventilation varies in each of 3 treatment groups

# **STUDY DESIGN**

**General**

This is a single center, open label, randomized, controlled, parallel group, clinical trial that will be conducted at Loghman Hakim Medical Education Center in conjunction with Shahid Beheshti University of Medical Sciences.

Eligible patients with confirmed SARS-Cov-2 infections will be randomly assigned in a 1:1:1 ratio to one of the three following therapeutic regiments: 1) IFNβ1a (Recigen) (Subcutaneous injections of 44μg (12,000 IU) on days 1, 3, 6) + Hydroxychloroquine + Lopinavir/Ritonavir (Kaletra) [IFNβ1a group], 2) IFNβ1b (Ziferon) (Subcutaneous injections of 0.25mg (8,000,000 IU) on days 1, 3, 6) + Hydroxychloroquine + Lopinavir/Ritonavir (Kaletra) [IFNβ1b group], and 3) Hydroxychloroquine (Single dose of 400 mg on day 1, orally, in all three arms) + Lopinavir/Ritonavir (Kaletra) (400mg/100 mg twice a day for 10 days, orally, in all three arms) [control group]. All three groups will receive standards of care consisting of the necessary oxygen support, non-invasive, or invasive mechanical ventilation.

Patients will be randomly allocated to three therapeutic arms using permuted, block-randomization to balance the number of patients allocated to each group. The permuted block (three or six patients per block) randomization sequence will be generated, using Package ‘randomizeR’ in R software version 3.6.1. and placed in individual sealed and opaque envelopes by the statistician. The investigator will enroll the patients and only then open envelopes to assign patients to the different treatment groups. This method of allocation concealment will result in minimum selection and confounding biases.

The present research is open-label (no masking) of patients and health care professionals who are undertaking outcome assessment of the primary outcome - time to clinical improvement.

## **Safety Assessments**

Regarding safety concerns, daily monitoring for adverse effects (AEs) and treatment-related AEs, vital signs, and laboratory testing will be carried out. All adverse effects should be recorded and include time, severity, symptoms, and their relation with aforementioned drugs.

## **Efficacy Assessments**

**Study Outcomes**

**Primary Outcome:**

Our primary outcome measure will be TTCI, defined as the time from enrollment to discharge from the hospital or a decline of two steps on the seven-step ordinal scale; Which so ever came first. Originally introduced by Beigel and colleagues in a posthoc analysis of an influenza study as a six-step ordinal scale, and currently recommended by the WHO R&D Blueprint Team (Accessed May 15, 2020, at https://www.who.int/teams/blueprint/covid-19.) for COVID-19 studies as a nine- step ordinal scale, the utilized seven-step ordinal scale consists of the subsequent categories: (I) Not hospitalized, and has no activity limitations; (II) Not hospitalized, but has activity limitations; (III) Hospitalized, but does not need any supplemental oxygen; (IV) Hospitalized, and needs supplemental oxygen; (V) Hospitalized, and needs either High-Flow Nasal Cannula (HFNC) or non-invasive ventilation; (VI) Hospitalized, and needs invasive ventilation; and (VII) Dead (9).

**Secondary Outcomes:**

Secondary outcomes include mortality from the date of randomization until day 21, by which all of the patients will have at least one of the following outcomes: 1) A decline of two steps on the seven-step ordinal scale, 2) Hospital discharge or 3) Death; SpO2 improvement defined as the difference between the last and the first recorded measurement during the hospitalization, using pulse-oximetry; length of stay in the hospital until the date of discharge from hospital or death from any cause, whichsoever came first; incidence of new mechanical ventilation uses from the date of randomization until day 21. Follow-ups of discharged patients will be done utilizing telemedicine visits, online, or over the telephone.

## **Study Drugs**

- **Hydroxychloroquine:**

400mg bid for first day then 200mg bid for 10 days

- **lopinavir/ritonavir (Kaletra):**

400mg/100mg bid for 10-14 days

For those that could not get orally:

5-ml suspension bid for 10-14 days

- **Interferon beta-1a:**

SC injection 44 micro-gram for 3 days (on days 1,3,6)

- **Interferon beta-1b:**

SC injection 0.25 mg for 3 days (on days 1,3,6)

## **Statistical Methods**

Statistical analysis will be performed by R version 3.6.1 software. The total sample size was calculated according to the Latouche and colleagues approach for estimating sample size in survival analysis with 80% power, alpha=0.05, Hazard Ratio (HR) of 3.0 (as the ratio of the hazard rates of time to clinical improvement (TTCI) corresponding to the intervention group compared to the control group) and assuming that 80% of patients would reach the primary outcome (10). The calculations will be carried out using Package ‘powerSurvEpi’ in R and accounted for a dropout

rate of 15%. Overall 60 patients, will be needed for the study, 20 for each arm.

Frequency rates and percentages will be used for categorical variables, and Interquartile Ranges (IQRs) and median will be used for continuous variables. Kruskal-Wallis test will be used for comparing the continuous variables. The Wilcoxon signed-rank test will compare the before and after intervention effects. Moreover, Chi-Square test will compare the frequency of categorical variables. We will use Kaplan–Meier to analyze the TTCI (compared with a log-rank test).

# **STUDY POPULATION**

##

## **Participants**

Sixty COVID-19 confirmed cases (using the RT-PCR test) will be enrolled in the trial between April 9^th^ to April 14^th^ 2020. Sixty patients will be randomly assigned in a 1:1:1 ratio to one of the three following therapeutic regiments; 20 were assigned to the IFNβ1a group; 20 were appointed to the IFNβ1b group, and the remaining 20 were assigned

to the control group.

## **Inclusion Criteria**

- Age ≥ 18
- COVID-19 Confirmed Cases by Means of RT-PCR
- Oxygen saturation (SPO2) ≤ 93% OR respiratory rate ≥ 24
- At least one of the following: Calibrated contactless infrared forehead thermometry temperature of ≥37.8, cough, sputum production, nasal discharge, myalgia, headache or fatigue on admission.
- Time of onset of the symptoms should be acute (Days ≤ 14)

## **Exclusion Criteria**

- Refusal to participate expressed by patient or legally authorized representative if they are present
- Patients with prolonged QT or PR intervals, Second or Third Degree heart block, Arrhythmias including torsade de pointes
- Patients using drugs with potential interaction with Hydroxychloroquine + Lopinavir/Ritonavir, Interferon-β 1a، Interferon-β 1b.
- Pregnant or lactating women.
- History of alcohol or drug addiction in the past 5 years.
- Blood ALT/AST levels > 5 times the upper limit of normal on laboratory results.

## **Drop Out**

In the event of developing an allergic reaction to the drug or the patient's refusal to cooperate, the participant will be considered as a dropout. Any other reason resulting in discontinuation of the therapeutic regiments would also be considered as a dropout.

# **RESTRICTIONs**

Our study has several limitations. The trial will be open-label and without a placebo-control group, which opens the possibility for risks of bias. Our trial will be carried out in a limited resource setting, where we have no access to the follow-up RT-PCT testing and quantitative Real-Time RT-PCR; therefore, therefore we could not determine the time to a negative RT-PCR test and the viral loads to shed further light on the effect of the studied drugs on viral dynamics. Finally, we will only enroll the severe patients with lower probabilities of survival; hence our findings cannot be extrapolated to all COVID-19 patients.

# **ETHICAL CONCIDERATION**

- Before conducting present study, the approval of the ethics committee and the research council of Shahid Beheshti University of Medical Sciences will be obtained.
- All research steps will be recorded in IRCT.
- Written consent is obtained from all patients before entering the study.
- We will provide patients with complete and clear information about the research process.
- At each phase of the study, patients are allowed to leave the study.
- The medical record of all patients are fully preserved

**REFERENCES**

1. Hageman JR. The coronavirus disease 2019 (COVID-19). Pediatric annals. 2020;49(3):e99-e100.

2. Lu R, Zhao X, Li J, Niu P, Yang B, Wu H, et al. Genomic characterisation and epidemiology of 2019 novel coronavirus: implications for virus origins and receptor binding. The Lancet. 2020;395(10224):565-74.

3. Chen Y, Liu Q, Guo D. Emerging coronaviruses: genome structure, replication, and pathogenesis. Journal of medical virology. 2020;92(4):418-23.

4. Spiegel M, Pichlmair A, Mühlberger E, Haller O, Weber F. The antiviral effect of interferon-beta against SARS-coronavirus is not mediated by MxA protein. Journal of clinical virology. 2004;30(3):211-3.

5. Channappanavar R, Perlman S, editors. Pathogenic human coronavirus infections: causes and consequences of cytokine storm and immunopathology. Seminars in immunopathology; 2017: Springer.

6. Zhou Y, Hou Y, Shen J, Huang Y, Martin W, Cheng F, et al. Network-based Drug Repurposing for Human Coronavirus. medRxiv. 2020.

7. Mosaddeghi P, Negahdaripour M, Dehghani Z, Farahmandnejad M, Moghadami M, Nezafat N, et al. Therapeutic approaches for COVID-19 based on the dynamics of interferon-mediated immune responses. 2020.

8. Cinatl J, Morgenstern B, Bauer G, Chandra P, Rabenau H, Doerr H. Treatment of SARS with human interferons. The Lancet. 2003;362(9380):293-4.

9. Beigel JH, Tebas P, Elie-Turenne M-C, Bajwa E, Bell TE, Cairns CB, et al. Immune plasma for the treatment of severe influenza: an open-label, multicentre, phase 2 randomised study. The Lancet Respiratory Medicine. 2017;5(6):500-11.

10. Latouche A, Porcher R, Chevret S. Sample size formula for proportional hazards modelling of competing risks. Statistics in medicine. 2004;23(21):3263-74.
