# Supplementary material for: Role of interferon therapy in severe COVID-19: the COVIFERON randomized controlled trial
Source: Sci Rep. 2021 Apr 13;11:8059. doi: 10.1038/s41598-021-86859-y (PMC8044200; doi:10.1038/s41598-021-86859-y)
Supplement: Supplementary file 3 — Supplementary Information 3. [file 41598_2021_86859_MOESM3_ESM.docx]

**Role of Interferon Therapy in Severe COVID-19: The COVIFERON Randomized Controlled Trial**

Ilad Alavi Darazam, MD ^1,2^, Prof. Shervin Shokouhi, MD ^1,2^, Mohamad Amin Pourhoseingholi, PhD ^3^, Seyed Sina Naghibi Irvani, MD, MPH, MBA ^1*^, Majid Mokhtari, MD ^4^, Minoosh Shabani, MD^1,2^, Mahdi Amirdosara, MD ^5^, Parham Torabinavid, MD ^6^, Maryam Golmohammadi, MD ^1^, SayedPayam Hashemi, MD ^1^, Arsalan Azimi, MD ^7^, Mohammad Hossein Jafarazadeh Maivan, MD ^8^, Prof. Omidvar Rezaei, MD ^9^, Prof. Alireza Zali, MD ^10^, Mohammadreza Hajiesmaeili, MD ^5^, Hadiseh Shabanpour Dehbsneh, MD ^1,2^, Akram Hoseyni Kusha, MD ^1,2^, Maryam Taleb Shoushtari, MD ^1,2^, Negar Khalili, MD ^1,2^, Azam Soleymaninia, MD ^1,2^, Prof. Latif Gachkar, MD ^1,2^, Ali Khoshkar, MD ^11^. [SBMU Taskforce on The COVIFERON Study]

**^1^** Department of Infectious Diseases, Loghman Hakim Hospital, Shahid Beheshti University of Medical Sciences, Tehran, Iran.

**^2^** Infectious Diseases and Tropical Medicine Research Center, Shahid Beheshti University of Medical Sciences, Tehran, Iran.

**^3^** Basic and Molecular Epidemiology of Gastrointestinal Disorders Research Center, Research Institute for Gastroenterology and Liver Diseases, Shahid Beheshti University of Medical Sciences, Tehran, Iran.

**^4^** Department of Pulmonary and Critical Care Medicine, Loghman Hakim Hospital, Shahid Beheshti University of Medical Sciences, Tehran, Iran.

**^5^** Anesthesiology Research Center Loghman Hakim Hospital, Shahid Beheshti University of Medical Sciences, Tehran, Iran.

**^6^** Student Research Committee, Hamadan University of Medical Sciences, Hamadan, Iran.

**^7^** Shiraz University of medical sciences, Zand Blvd., Shiraz, Iran.

**^8^** Clinical Research Development Unit, Bohlool Hospital, School of Medicine, Gonabad University of Medical Sciences, Gonabad, Iran.

**^9^** Skull Base Research Center, Loghman Hakim Hospital, Shahid Beheshti University of Medical Sciences, Tehran, Iran.

**^10^** Functional Neurosurgery Research Center, Shohada Tajrish Neurosurgical Center of Excellence, Shohada Tajrish Hospital, Shahid Beheshti University of Medical Sciences, Tehran, Iran.

**^11^** Department of Surgery, Loghman Hakim Hospital, Shahid Beheshti University of Medical Sciences, Tehran, Iran.

**^*^Corresponding Author:**

Seyed Sina Naghibi Irvani, MD, MPH, MBA,
Department of Infectious Diseases, Loghman Hakim Hospital, Shahid Beheshti University of Medical Sciences, Tehran, Iran. (**Email:** Sina.Irvani@sbmu.ac.ir)
**P.O. Box:** 1567812907, **Tel:** +98-914-1182825, **Fax:** +98-413-3845238

Statistical Analysis Plan (SAP)

**Project:**
Effectiveness of Interferon Beta 1a, Compared to Interferon Beta 1b And the Usual Therapeutic Regimen to Treat Adults with Moderate to Severe COVID-19

**Principal Investigator:**
Seyed Sina Naghibi Irvani, MD, MPH, MBA

**Clinical Trial identifier:**
NCT04343768 (National Institutes of Health Clinical trials; www.clinicaltrials.gov)

**Author:** Mohamad Amin Pourhoseingholi, PhD

Associate Professor of Biostatistics

Shahid Beheshti University of Medical Sciences

Taleghani Hospital, Tehran, Iran

[Pourhoseingholi@sbmu.ac.ir](mailto:Pourhoseingholi@sbmu.ac.ir)

**Abbreviations:**

CI: Confidence Interval

IQR: Interquartile Range

ITT: Intention-to-Treat

HR: Hazard Rate

MITT: Modified Intention-to-Treat

MI: Multiple Imputation

RCT: Randomized Clinical Trial

SAP: Statistical Analysis Plan

1. **Introduction**

The aim of this project is to test in a single-center randomized clinical trial (RCT), if Interferon beta-1a and Interferon beta 1b would be more efficient in treating COVOD-19 in Iranian patients, compare to standard care.

This statistical analysis plan (SAP) will provide more details regarding this RCT and the corresponding analyses behind it.

1. **Study design**

Study subjects will be recruited from Loghman Hakim Hospital (a University Hospital in Tehran, Iran). The patients with COVID-19, which confirmed using the RT-PCR test with the following eligibility criteria: ≥ 18 years of age AND (Oxygen saturation (SPO2) ≤ 93% OR respiratory rate ≥ 24) AND at least one of the following: Contactless infrared forehead thermometer temperature of ≥37.8, cough, sputum production, nasal discharge, myalgia, headache or fatigue on admission, and time of onset of the symptoms should be acute (Days ≤ 14).

The study is a parallel-group RCT with three arms during maximum of 28 days. Subjects will be randomized to the standard care plus Interferon beta-1a, standard care plus Interferon beta 1b and control group (standard care only). Eligible patients will randomly assigned in a 1:1:1 ratio to receive either Interferon Beta 1a, Interferon Beta 1b or standard care only. The permuted block (three or six patients per block) randomization sequence was performed and randomization has been done using Package ‘randomizeR’ in R software version 3.6.1. and placed in individual sealed and opaque envelopes.

**Figure 1. Flowchart of screening and inclusion process.**

- 1. **Sample size calculation**

The primary outcome measure for the power calculation is the hazard rates of clinical improvement corresponding to the treatment group compare to standard care group. The total sample size was calculated according to Latouche et al (2004) approach for estimating sample size in survival analysis (ref) with 80% power, alpha = 0.05, hazard ratio of 3.0 (as the ratio of the hazard rates of clinical improvement corresponding to the pooled treatment groups compare to standard care group) and assuming that 80% of patients would reach clinical improvement. The calculations were carried out using Package ‘powerSurvEpi’ in R, and accounting for dropout rate of 15%, 60 patients should be recruited for this trial, (exact 20 patients in each arm).

1. **Aims and objectives**

To investigate the effectiveness of Interferon Beta 1a, compared to Interferon Beta 1b and the usual therapeutic regimen in COVID-19 in patients that have tested positive and are moderately to severely ill.

1. **Outcomes**
   1. **Primary outcome**

Time to clinical improvement is the primary outcome measure. This is an improvement of two points on a seven-category ordinal scale (recommended by the World Health Organization: Coronavirus disease (COVID-2019) R&D. Geneva: World Health Organization) or discharge from the hospital, whichever came first.

- 1. **Secondary outcomes**

Secondary outcomes include mortality from the date of randomization until the last day of study, SpO2 Improvement, Duration of hospitalization from date of randomization until the date of hospital discharge or date of death from any cause, whichever came first, incidence of new mechanical ventilation uses from date of randomization, Serious adverse effects and other blood laboratory parameters (WBC, Lymphocyte count, Neutrophil count, Platelet count, Serum creatinine, etc.).

- 1. **Safety outcomes**

Adverse events will be reported during hospitalization.

1. **Populations be analyzed**

**Intention-to-treat (ITT):** All randomized study subjects. This will be seen as the primary population for the analysis.

**Modified Intention-to-treat (MITT) or Per-Protocol Analysis:** The population will be revised (if needed) based on-treatment’ analyses.

1. **Analyses**

All outcomes will be presented using descriptive statistics; normally distributed data by the mean and standard deviation (SD) and if they would not meet the normality, the median and interquartile range (IQR) will be presented. Categorical variables will be presented using counts and percentages. Statistical analysis will be performed by R version 3.6.1 software.

- 1. **Primary outcome**

The primary analysis will compare intervention groups according to changing in the time to clinical improvement. The comparison will be done by Kaplan–Meier plot and log-rank test. The Hazard ratios with

95% confidence intervals will be calculated by the Cox proportional-hazards model.

- 1. **Secondary outcomes**

Secondary outcomes with normal distribution will be analysis using ANOVA, if the distribution will not normal or due to sample size there is a possibility to decreasing the power, non-parametric tests including Kruskal-Wallis test or Median test would be performed. For testing the SpO2 Improvement, paired sample T-test (or non-parametric Wilcoxon signed-rank test) will be performed.

1. **Missing data**

For ITT population analysis, in case of missing for primary outcomes, model based multiple imputation (MI) will be used.

For secondary outcomes, the approach could be depends on the importance of the outcomes and the causes of missing. So, both multiple imputation (MI) and analysis based on available data could be assessed.
